# Low-Temperature Performance and Damage Constitutive Model of Eco-Friendly Basalt Fiber–Diatomite-Modified Asphalt Mixture under Freeze–Thaw Cycles

**DOI:** 10.3390/ma11112148

**Published:** 2018-10-31

**Authors:** Yongchun Cheng, Di Yu, Guojin Tan, Chunfeng Zhu

**Affiliations:** College of Transportation, Jilin University, Changchun 130025, China; chengyc@jlu.edu.cn (Y.C.); yudi16@mails.jlu.edu.cn (D.Y.); zcf-mine@163.com (C.Z.)

**Keywords:** diatomite, basalt fiber, asphalt mixture, low-temperature, damage constitutive model

## Abstract

Asphalt pavement located in seasonal frozen regions usually suffers low-temperature cracking and freeze–thaw damage. For this reason, diatomite and basalt fiber were used to modify asphalt mixtures. An indirect tensile test was used to determine the low-temperature performance of the asphalt mixture. The influences of freeze–thaw (F–T) cycles on strength, tensile failure strain, stiffness modulus, and strain energy density were analyzed. The variation of the stress–strain curve under F–T cycles was analyzed. The stress–strain curve was divided into a linear zone and nonlinear zone. The linear zone stress ratio and linear zone strain ratio were proposed as indexes to evaluate the nonlinear characteristics of the stress–strain curve. The results show that the basalt fiber–diatomite-modified asphalt mixture had better low temperature crack resistance and antifreeze–thaw cycles capacity compared to the control asphalt mixture. The F–T cycles made the nonlinear characteristics of the stress–strain relationship of the asphalt mixture remarkable, and also decreased the linear zone stress ratio and linear zone strain ratio. The damage constitutive model established in this paper can describe the stress–strain relationship after F–T damage well.

## 1. Introduction

The asphalt concrete pavement in seasonal frozen regions is affected by low temperature and freeze–thaw (F–T) cycles. Under low-temperature conditions, the pavement is prone to thermal cracking. Under loading and F–T cycles, cracks will develop rapidly, forming pavement distresses and damage, such as stripping, potholes, and surface deterioration, seriously effecting the service life of the road. To reduce the effect of F–T cycles on the low-temperature property of the asphalt mixture, more addition can be used to improve the low-temperature performance and frost resistance of the mixture in seasonal frozen regions.

Diatomite is a material with high porosity, high surface absorption rate, abundant resources, and low cost [1,2]. It has been widely utilized to modify asphalt, asphalt binders, and asphalt mixtures. Cheng et al. [3] demonstrated the anti-aging properties of diatomite-modified asphalt. The results indicated that diatomite is useful in improving the high-temperature stability and anti-aging properties of asphalt. Cong et al. [4,5] studied diatomite-modified asphalt binders. The results indicated that no chemical reaction occurs between asphalt and diatomite. The high-temperature properties and anti-aging properties are elevated by the addition of diatomite, while the low-temperature properties of asphalt binder are not sufficiently improved. Yang et al. [6] revealed that the addition of diatomite has a great effect on improving the high-temperature performance, fatigue performance, and moisture resistance of the asphalt mixture, but has little effect on the improvement of its low-temperature performance. However, the low-temperature performance of asphalt mixtures is very important in seasonal frozen regions, such as Jilin province in China. Therefore, it is a feasible method to deal with these distresses of diatomite-modified asphalt mixtures by adding other modifiers.

Basalt fiber (BF) is an eco-friendly mineral fiber for reinforcing the properties of asphalt mastic and asphalt mixtures that exhibits high strength, low water absorption, stable chemical characteristics, high resistance to temperature, less waste in production and no harm to the environment after abandonment [7]. Much research has investigated the effect of BF on asphalt concrete in recent years. Zheng et al. [8] evaluated the low-temperature bending property and fatigue property of basalt fiber-modified asphalt mixtures under chloride erosion and F–T cycles. The results reveal that the ultimate tensile strength and the maximum bending strain can be improved by the addition of BF. Using BF to modify asphalt mixtures can improve resistance to low temperature, fatigue, and chloride erosion and F–T action. Qin et al. [9] investigated the characterization of asphalt mastics modified by BF. The results reveal that a stable three-dimensional network would be formed in asphalt mastic and result in the improvement of the crack resistance performance by the addition of BF. Zhang et al. [10,11] studied the impact of BF distribution on the asphalt mortar through a new three-dimensional fiber distribution model. The results show that BF can effectively improve the flexural–tensile strain. Liu et al. [12] studied the variation and influence of fibers on the low-temperature performance of asphalt mastic and asphalt mixtures though the bending beam rheometer (BBR) test and low-temperature beam bending test. The results reveal that the addition of BF brings a lower creep stiffness and higher creep rate for asphalt mastic at low temperature. Gao [13] studied the performance of basalt fiber modified asphalt mixtures though low-temperature IDT test. The results show that that the addition of BF brings a higher failure strength and failure strain. Zhao [14] used the beam bending test and beam bending creep test to evaluate low-temperature performance of basalt fiber modified asphalt mixtures. The results show that the flexural strength, failure strain and creep rate were increased by adding basalt fiber, and the low-temperature cracking resistance can be improved. In summary, the low-temperature properties and crack resistance of asphalt mixtures can be improved by adding a suitable content of BF.

The effects of combination diatomite and basalt fiber on asphalt mixtures are still unclear. Cheng et al. [15] studied the properties of basalt fiber–diatomite asphalt mastic by softening point, cone penetration, viscosity, and dynamic shear rheological (DSR) tests. The results show that the high and low-temperature properties of basalt asphalt mastic were improved by the addition of basalt fiber–diatomite. Arash Davar et al. [16] studied the properties of basalt fiber–diatomite asphalt mixtures through a four-point bending beam test and indirect tensile strength test. The results show that the fatigue resistance and low-temperature properties of basalt fiber–diatomite asphalt mixtures had been improved. If the disadvantage of diatomite-modified mixtures can be solved by basalt fiber, then the basalt fiber–diatomite-modified asphalt mixture can be well-used in seasonal frozen regions.

F–T cycles have a great influence on the pavement of seasonal frozen regions. Many researchers investigated the effect of F–T cycles on asphalt mixtures through laboratory experiments, such as the indirect tensile (IDT) test, beam bending test, Marshall stability tests, fatigue tests, complex modulus test, and so on [17,18,19,20,21]. The engineering character of asphalt mixtures would be reduced due to the damage caused by water-freeze expansion, and the weakened bonding between mortar and aggregate under F–T cycles. The low-temperature indirect tensile test is one of the simplest tests to evaluate the low-temperature properties of asphalt mixtures [22,23], which can be adopted to analyze the modification effect of basalt fiber and diatomite on asphalt mixture under F–T cycles.

As for the effects of the F–T cycles and modifier on the change in the stress–strain curve for asphalt mixtures, few relevant studies have been published [24,25]. The statistical damage constitutive model is widely used for rock and soil under F–T and loading [26,27]. By putting forward some indexes to describe the nonlinear change of the stress–strain curve and finally establishing the damage constitutive model of basalt fiber–diatomite-modified asphalt mixtures under F–T cycles, we can describe and analysis effect of F–T cycles and modifier on the asphalt mixture at low temperature.

In this paper, a diatomite-modified asphalt mixture (DAM), basalt fiber-modified asphalt mixture (BFAM), and diatomite–basalt fiber-modified asphalt mixture (DBFAM) were studied and compared with a matrix asphalt mixture (AM). The F–T cycles were used to simulate the climatic conditions of the seasonal frozen regions. The indirect tensile test was used to determine the low-temperature performance of the asphalt mixture. The influence of the F–T cycles on IDT strength, IDT failure strain, failure stiffness modulus, and strain energy density were analyzed. The variation of the stress–strain curve under F–T cycles was analyzed. The linear zone stress ratio and linear zone strain ratio were used as indexes to evaluate the nonlinear characteristics of the stress–strain curve. A statistical damage constitutive model was established to describe the stress–strain relationship after F–T damage.

## 2. Materials and Methods

### 2.1. Raw Materials

Base asphalt AH-90, from the Panjin petrochemical industry, in Panjin City, Liaoning Province of China, was used as the binder. The physical indexes of the base asphalt are listed in Table 1.

**Figure 1:**
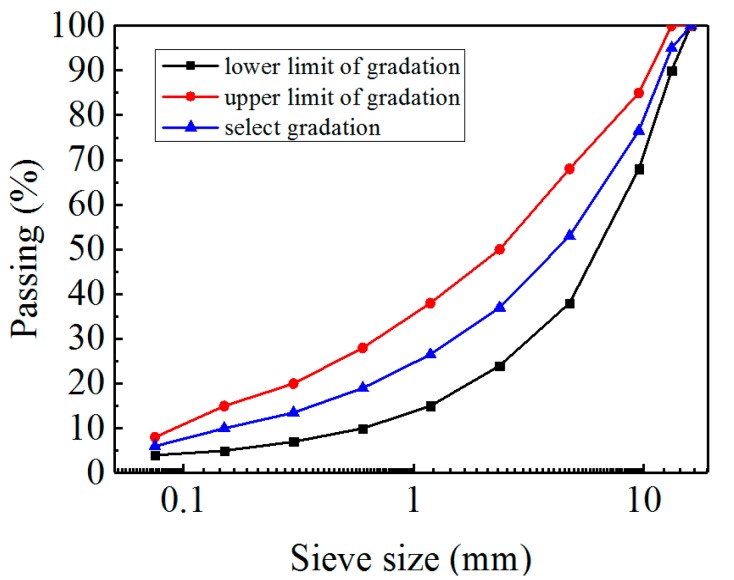
The Grading Curve aggregates used in this study.

The aggregates were basalt and the mineral filler was limestone powder. The physical properties of basalt aggregate and mineral filler are listed in Table 2 and Table 3, correspondingly. The gradation was designed with 13.2 mm nominal maximum size. The selected gradation of the asphalt mixture is shown in Figure 1.

Diatomite was produced by Changchun Diatomite Products Co., Ltd., in Linjiang City, Jilin Province, China. Its physical properties are presented in Table 4.

Basalt fiber is the short fiber from Jiuxin Basalt Fiber Inc., in Jilin City, Jilin Province, China, and its properties are presented in Table 5.

### 2.2. Specimen Preparation

Cylindrical specimens (101.6 ± 0.2 mm diameter by 63.5 mm ± 1.3 mm high) for each mixture were prepared using a Marshall compactor. The specimens were compacted in a Marshall Compactor with 75 beats on each side. All of them were prepared with the same gradation at optimum asphalt content (OAC). The OAC of the asphalt mixture was obtained by applying the Marshall Methods [28]. Three identical samples were used to investigate low-temperature cracks under F–T cycles in asphalt mixes modified with basalt fibers and diatomite powder. According to previous research results [29], the proportions of the diatomite–basalt compound-modified asphalt mixture and optimum asphalt content are determined as follows:

- The control asphalt mixture sample (with no additives) (AM) has an OAC of 4.78%.
- The diatomite (corresponding to the volume ratio of the diatomite to entire filler is 6.5%) modified asphalt mixture (DAM) has an OAC of 5.12%.
- The basalt fiber (0.25% by weight of the asphalt mixture) modified asphalt mixture (BFAM) has an OAC of 5.09%.
- The basalt fiber (0.25%) and diatomite (6.5%) compound modified asphalt mixture (DBFAM) has an OAC of 5.22%.

The preparation procedures of DBFAM are as follows. Aggregates were mixed in the mixing oven at 160 °C. The basalt fiber was added with aggregates; they were mixed for about 60 s to disperse the basalt fiber evenly in the aggregates. Then, the asphalt was added, and they were mixed for about 90 s to make the aggregate surface uniformly coated by asphalt. Then, the mineral filler and diatomite was added and the diatomite and basalt fiber compound modified asphalt mixture was obtained after a second mix of 90 s. Except for the addition of modified materials, the preparation procedures of AM, BFAM, DAM are similar to that of DBFAM. The mixing temperature and the mixing time of each step are the same as that of DBFAM.

### 2.3. Process of Freeze–Thaw

Before the test, every specimen was immersed into water and under a vacuum 98.0 kPa for 15 min and soaked in atmospheric pressure for 30 min. Each cycle consisted of freezing at −18 °C for 16 h, followed by soaking in water at 60 °C for 8 h. Before freezing, the specimen was placed into a plastic bag with 10 mL water. Before thawing, the specimens were removed from the plastic bags. After 0, 3, 6, 9, 12, and 15 F–T cycles, the low-temperature indirect tensile test was carried out.

### 2.4. Low-Temperature Indirect Tensile Test

The low-temperature tensile property is a significant index to evaluate the crack resistance for asphalt mixture pavement, which is often investigated using the three-point bending method and indirect tensile method [30,31]. Guo et al. regarded that, while using the bending test to evaluate the crack resistance of diatomite–glass fiber-modified asphalt mixture, the effect of the modifier could not be well reflected because there were fewer fibers and less diatomite at the bottom of the middle span [1]. Therefore, the low-temperature indirect tensile test was used to evaluate the crack resistance of the DBFAM.

The specimens after the process of freeze–thaw were tested at a temperature of −10 °C with an environment box. Before the test, the specimens were placed in a chamber at −10 °C for 5 h to reach thermal equilibrium. The constant rate was 1 mm/min [28]. During the test, the vertical deformation on the top surface of the specimen and the load could be recorded by computer.

The IDT strength, $R_{T}$, the IDT failure strain $\varepsilon_{T}$, and the failure stiffness modulus $S_{T}$ can be calculated by following equations [28]:

(1) $$R_{T}=0.006287P_{T}/h\;$$

(2) $$\varepsilon_{T}=X_{T}\times (0.0307+0.0936\mu )/(1.35+5\mu )\;$$

(3) $$S_{T}=P_{T}\times (0.27+1.0\mu )/(h\times X_{T})\;$$

(4) $$X_{T}=Y_{T}\times (0.135+0.5\mu )/(1.794-0.0314\mu )\;$$

where $R_{T}$ is the indirect tensile strength, MPa; $\varepsilon_{T}$ is the tensile failure strain; $S_{T}$ is the failure stiffness modulus, MPa; $P_{T}$ is the indirect tensile failure load, N; $Y_{T}$ is the vertical deformation, mm; $X_{T}$ is the horizontal deformation, mm; μ is the Poisson ratio, which is 0.25 in this test; and h is the height of specimen, mm.

However, the conclusions obtained from $R_{T}$, $\varepsilon_{T}$, and $S_{T}$ may be inconsistent sometimes. Thus, the deformation energy index was selected to evaluate the low-temperature performance as a comprehensive index [6]. The process of low-temperature cracking for the asphalt mixture is a process of dissipation. The higher is the deformation energy, the stronger is the crack resistance at low temperature.

**Figure 2:**
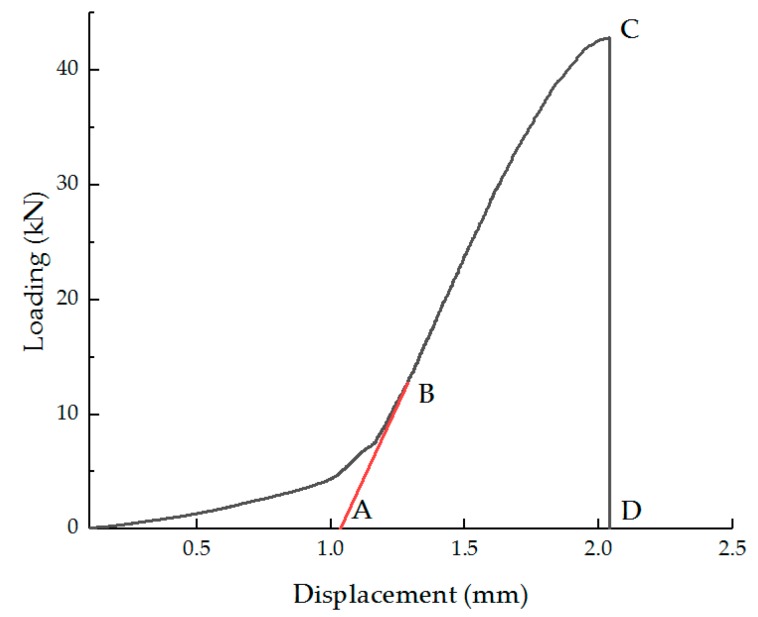
Load–deformation curve.

The load–displacement curve is modified in Chinese Standard Specification (JTG E20-2011), which extends the straight line segment and takes the intersection with the abscissa as the origin of the curve, as shown in Figure 2. The deformation energy can be defined as the area of the modified stress–strain curve. The deformation energy density can be calculated by Equation (5):

(5) $$Q_{B}=\frac{1}{h}\int_{Y_{T1}}^{Y_{T2}}P_{T}(y)dy\;$$

where $Q_{B}$ is the deformation energy density, N∙m; $Y_{T1}$ the origin displacement, mm; and $Y_{T2}$ the critical displacement, mm.

Those indexes were calculated for different asphalt mixtures under F–T cycles to analyze the change of the low-temperature performance under F–T cycles. To evaluate the F–T resistance of different asphalt mixtures, the loss ratio of each index before and after F–T cycles is given by Equation (6):

(6) $$\Delta I_{i}=\left(1-\frac{I_{i}}{I_{0}}\right)\times 100\;$$

where $\Delta I_{i}$ is the loss ratio of each index after 𝑖 times of F–T cycles, $I_{0}$ is without F–T cycles, and $I_{i}$ is each index after 𝑖 times of F–T cycles. If the index is increased under F–T cycles, then the change ratio of it can be used as $-\Delta I_{i}$.

### 2.5. The Nonlinear Evaluation Indexes of Stress–Strain Curves

**Figure 3:**
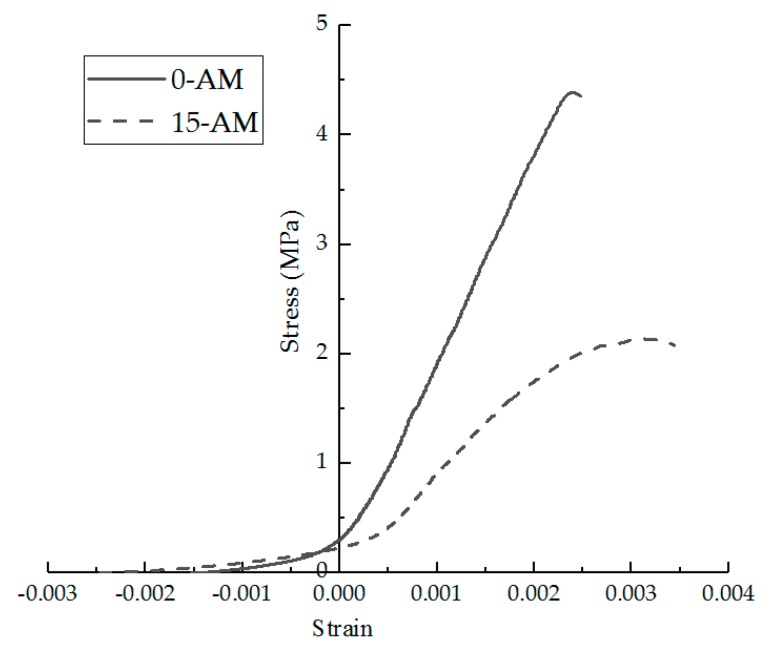
Stress–strain curve under freeze–thaw (F–T) cycles.

The stress–strain curve was calculated from the load–displacement curve collected by the testing machine. The stress was calculated as the $R_{T}$ in Equation (1). The strain was calculated as the $\varepsilon_{T}$ in Equation (2). The stress–strain curves of the asphalt mixture will change after F–T cycles. In the previous study, the researchers only focused on the failure strength of the asphalt mixture under the F–T cycles [18,21], but there was no description and evaluation index of the nonlinear change of the stress–strain curve after the F–T cycles. The stress–strain curves were calculated for AM under F–T cycles, as shown in Figure 3. As can be seen in Figure 3, the stress–strain curve of the asphalt mixture exhibits nonlinear softening characteristics under the action of F–T cycles. Asphalt mixtures are no longer similar to elastomers at low temperature under F–T cycles.

**Figure 4:**
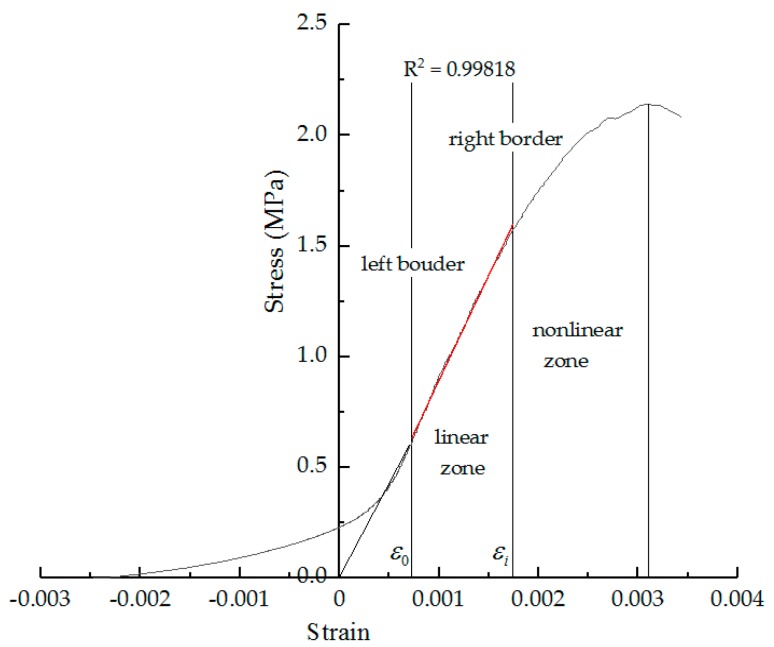
Linear zone and nonlinear zone in stress–strain curve.

Therefore, the stress–strain curve obtained by the low-temperature indirect tensile test of the asphalt mixture after F–T cycles is divided into a linear zone and a nonlinear zone in this paper. The linear fitting is performed through the straight line segment in the stress–strain curve to ensure that R^2^ is more than 0.998, to determine the linear zone slope and boundary point location. When R^2^ does not meet the accuracy requirement, it iteratively reduces the boundary of the zone and solves the end point of the straight segment, as shown in Figure 4. The specific steps are as follow:

Step 1: Set the left initial border of the line segment. This paper focuses on the end point of the straight line segment, so we can take the left end of the fitted area on the straight line segment. To simplify the calculation, the left end point of the fitted area is guaranteed to be on the straight line segment. Therefore, the left end point of the fitting zone is taken as the corresponding point of the 0.3 times stress peak, set ε = $\varepsilon_{0}$.

Step 2: Set the right initial border of the fitted area. The left end point of the fitting zone is taken as the corresponding point of the stress peak, set ε = $\varepsilon_{1}$.

Step 3: Determine whether the curve in the interval [$\varepsilon_{0}$, $\varepsilon_{i}$] (i = 1, 2, 3 … n) is a straight line. The curve in the interval [$\varepsilon_{0}$, $\varepsilon_{i}$] is linearly fitted by the least squares method. Determine whether the R^2^ is large enough. If R^2^ > 0.998, the interval can be seen as a linear zone, and Step 5 will be executed. Otherwise, the interval is still obtained as the nonlinear zone, so Step 4 will be executed.

Step 4: Reduce the right border of the fitted area. Calculate the biggest right intersection point between fitting straight line and curve, set ε=$\varepsilon_{i}$. Go back to Step 3.

Step 5: Output right border of the fitted area. Set $\varepsilon_{i}$ = $\varepsilon_{L}$. Set stress as $R_{L}$.

The linear zone stress ratio and the linear zone strain ratio are defined as indexes for describing the nonlinear problem under the F–T cycles. Therefore, the influence of different additives in asphalt mixtures on the two indexes under F–T cycles can be analyzed. The variation of the stress–strain curve under F–T cycles is analyzed. The linear zone stress ratio and linear zone strain ratio are used as indexes to evaluate the nonlinear characteristics of stress–strain curves.

The linear zone stress ratio ($RR_{L}$) is the ratio of the linear zone stress to $R_{T}$, which can be calculated by Equation (7). The linear zone strain ratio ($R\varepsilon_{L}$) is the ratio of the linear zone strain to $\varepsilon_{T}$, which can be calculated by Equation (8). These two indexes are proposed to describe the end point of the linear zone of the stress–strain curve.

(7) $$RR_{L}=R_{L}/R_{T}\;$$

(8) $$R\varepsilon_{L}=\varepsilon_{L}/\varepsilon_{T}\;$$

Through these two indexes, the full-stage stress–strain curve and mechanical properties of the asphalt mixture after F–T cycles can be better described.

The elastic stiffness modulus of linear zone can be obtained by using the two nonlinear indictors. The elastic stiffness modulus can exclude the influence of the nonlinear zone in the loading process; in other words, the modulus damage introduced by the loading damage and the coupling effect of loading and F–T cycles damage are eliminated. The loss ratio of elastic stiffness modulus is calculated as the damage only caused by F–T cycles.

(9) $$S_{nL}=k\cdot E_{n}=k\cdot \frac{\sigma_{L}}{\varepsilon_{L}}=\frac{R\varepsilon_{L}}{RR_{L}}\cdot S_{T}\;$$

(10) $$D_{nL}=1-S_{nL}/S_{0L}=1-E_{n}/E_{0}\;$$

where $S_{nL}$ is the elastic stiffness modulus of linear zone. $E_{n}$ is the slope of the stress–strain curve. k is calculated using Equations (1)–(4) as 1.721.

### 2.6. Damage Constitutive Model of the Asphalt Mixture under F–T Cycles

F–T cycles not only decrease the mechanical properties of the asphalt mixture, but also cause some internal damage, which causes a change to the constitutive relation of the asphalt mixture subjected to loading. Through the damage analysis of the different kinds of asphalt mixtures, we can establish the damage constitutive model, which reflects the deformation characteristics of asphalt mixtures subject to F–T cycles.

The statistical damage constitutive model is widely used for rock and soil under F–T cycles and loading [26,27]. According to Lemaitre’s stress equivalence principle (1985), an equivalent relation can describe the constitutive relation between effective stress and strain of the damaged material and undamaged one.

(11) $$\sigma =\sigma^{\prime }(1-D)=E\varepsilon (1-D)\;$$

The constitutive relationship of the asphalt mixture under F–T cycles at low temperatures can be described as follows:

(12) $$\sigma =\{\begin{array}{c} E_{n}\cdot \varepsilon \\ E_{n}(1-D_{t})\cdot \varepsilon \end{array}\begin{array}{cc} , & \left(0<\varepsilon \leq \varepsilon_{L}\right)\\ , & \left(\varepsilon_{L}<\varepsilon \right) \end{array}\;$$

where $E_{n}$ is the slope of straight line of linear zone. $D_{t}$ is defined to describe the damage caused by loading.

On this basis, the following assumptions are made for asphalt mixture at low temperature:

- The asphalt mixture at low temperature accords with the generalized Hooke’s law.
- The strength of micro-unit conforms to the statistical law of the three-parameter Weibull:

(13) $$\varphi \left(\varepsilon \right)=\frac{n}{m}(\varepsilon -\gamma {)}^{n-1}e^{\left[-\frac{(\varepsilon -\gamma {)}^{n}}{m}\right]}\;$$

where ε is the strain of the asphalt mixture and *n* and *m* are the physical and mechanical parameters of the asphalt mixture under external load, respectively. γ is the threshold parameter. Considering that the nonlinearity is caused by damage, the threshold is taken as $\varepsilon_{L}$.

The damage of the asphalt mixture at low temperature is caused by the uneven failure of the mixture micro-units. $D_{t}$ can be defined as the statistical damage variable, as the ratio of damaged micro-units to all the micro-units. Then, we can obtain the loading damage variable as follows:

(14) $$D_{t}=\int_{\gamma }^{\varepsilon }\varphi \left(x\right)dx=\int_{\gamma }^{\varepsilon }\frac{n}{m}(x-\gamma {)}^{n-1}e^{\left[-\frac{(x-\gamma {)}^{n}}{m}\right]}dx=1-e^{\left[-\frac{(\varepsilon -\varepsilon_{L}{)}^{n}}{m}\right]}\;$$

Substituting Equations (10) and (14) with Equation (12), the constitutive equation of the asphalt mixture at low temperature after F–T cycles under loading is as follows:

(15) $$\sigma =\{\begin{array}{c} (1-D_{nL})E_{0}\varepsilon \\ (1-D_{nL})E_{0}e^{\left[-\frac{(\varepsilon -\varepsilon_{L}{)}^{n}}{m}\right]}\varepsilon \end{array}\begin{array}{cc} , & \left(0<\varepsilon \leq \varepsilon_{L}\right)\\ , & \left(\varepsilon_{L}<\varepsilon \right) \end{array}\;$$

## 3. Results and Discussion

### 3.1. IDT Strength

**Figure 5:**
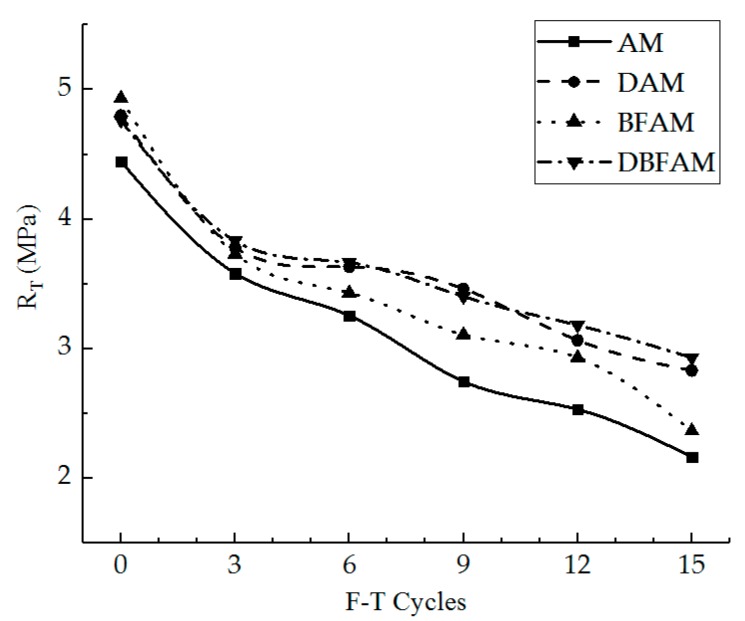
Indirect tensile (IDT) strength under F–T cycles. AM, Matrix asphalt mixture; DAM, diatomite-modified asphalt mixture; BFAM, basalt fiber-modified asphalt mixture; DBFAM, diatomite–basalt fiber-modified asphalt mixture.

**Figure 6:**
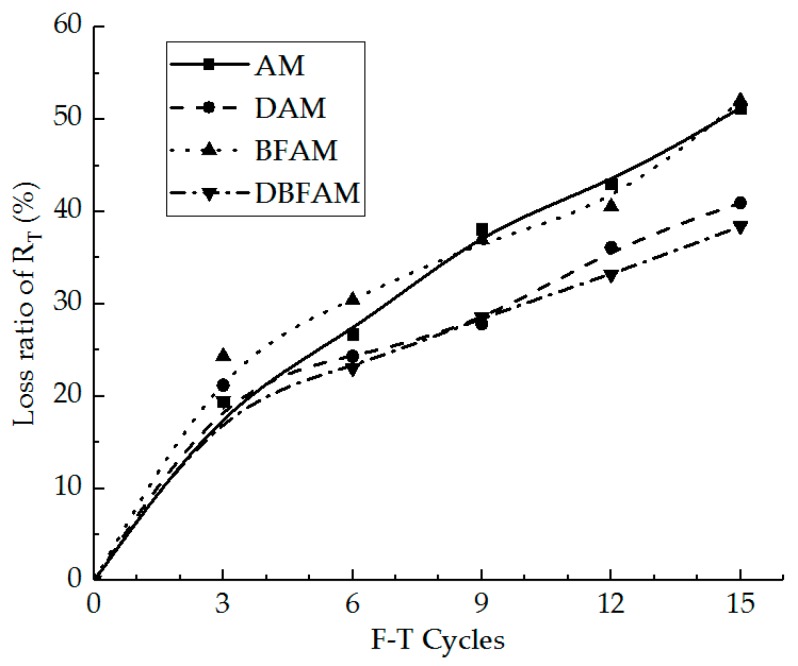
Loss ratio of IDT strength under F–T cycles.

IDT strength can reflect the failure peak stress of the asphalt mixture at low temperature. In Figure 5, we can see the IDT strength for the AM, BFAM, DAM, and DBFAM asphalt mixtures after F–T Cycles 0, 3, 6, 9, 12, 15. The loss ratio of IDT strength is shown in Figure 6.

As can be seen in Figure 5 and Figure 6, the IDT strength for each kind of asphalt mixture decreases and the loss ratio of IDT strength increases with the progress of F–T cycles. As the F–T cycles increase, the strength loss increases and the rate decreases more slowly. This indicates that the F–T cycle has a significant effect on the mechanical properties of the asphalt mixture in the early stage, and tends to change slowly after six F–T cycles. The reason lies in that the F–T actions will increase the air voids and cause micro-crack formation and propagation in the asphalt mixture. This is because F–T cycles weaken the bonding between binder and stone.

Meanwhile, the strengths of DAM, BFAM, and DBFAM are all higher than that of AM under F–T cycles. The loss of strength is significantly decreasing in DAM and DBFAM, while not significantly decreasing in BFAM compared with in AM. This indicates that adding basalt fiber can improve the strength and the diatomite can improved the resistance to F–T cycles. The influence mechanism basalt fiber on strength may be due to its reinforcing, toughening and preventing cracks and redistribution of stress in asphalt mixture. The diatomite modified asphalt can also improve the strength, which may be caused by the hardening effects of adsorption between diatomite and asphalt. Diatomite is a porous material with a large specific surface area. After it is added to the asphalt mixture, the bonding ability of the asphalt mortar is improved due to the hardening effects of adsorption between diatomite and asphalt, and the water is harder to invade the bonding place between aggregate and asphalt binder. Above all, composite-adding the two modified materials can improve both low-temperature performance and resistance to F–T cycles.

### 3.2. IDT Failure Strain

**Figure 7:**
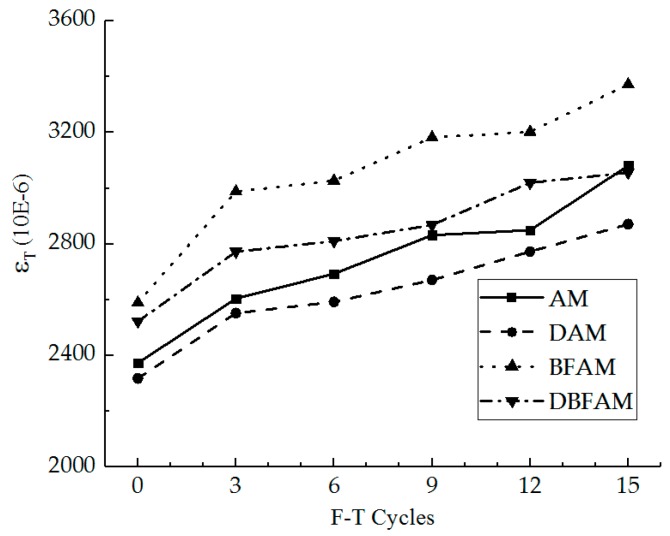
IDT failure strain under F–T cycles.

**Figure 8:**
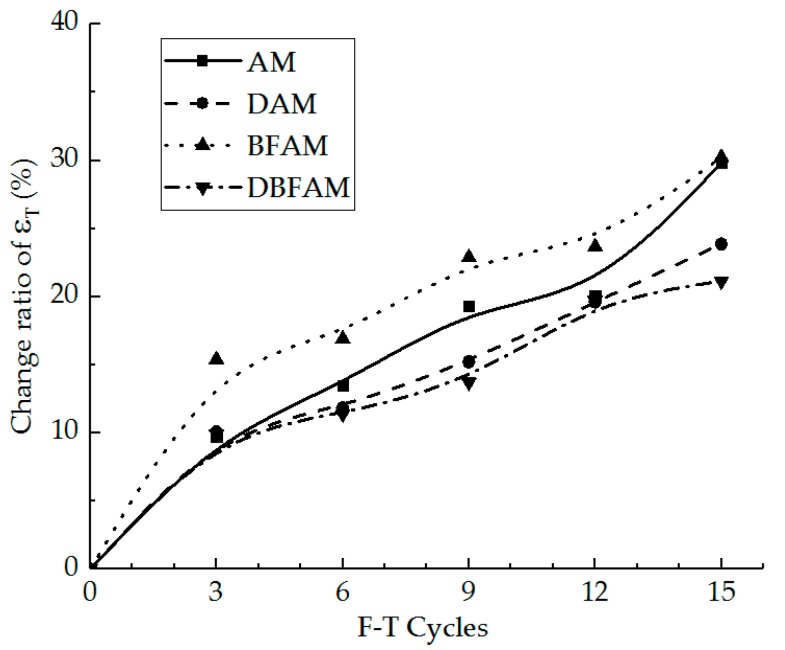
Change ratio of IDT failure strain under F–T cycles.

The IDT failure strain and the change ratio of it were calculated for different specimens under F–T cycles, as shown in Figure 7 and Figure 8.

As can be seen in Figure 7 and Figure 8, the results of IDT failure strain on each kind of asphalt mixture increase and the growth ratio also increases under F–T cycles. As the F–T cycles increase, the failure strain increases and the change rate become slow. This indicates that the F–T cycles have a significant effect on the deformation properties of the asphalt mixture in the early stage, and tend to be changed slowly after six F–T cycles.

Before the F–T cycles, the failure strains of BFAM and DBFAM are higher than that of AM, while that of DAM is less than that of AM. This indicates that adding basalt fiber can improve the failure strain, while adding diatomite is not significant. According to previous research, the failure strain would decrease through the addition of diatomite [1,16], and increase through the addition of basalt fiber [32]. The result of DBFAM reflects that adding basalt fiber can solve the problem of diatomite-modified asphalt mixture at low temperature.

Under F–T cycles, the failure strain of BFAM increases remarkably compared with that of others. The reason is that water may be easier to invade in the combination of mortar and stone through fiber in BFAM under F–T cycles. As shown in the failure strain of DAM and DBFAM, adding diatomite can solve the problem in F–T cycles by enhancing adhesion and water stability.

Above all, composite-adding the two modified materials is suitable for asphalt mixtures at low temperature under F–T cycles. It can give full play to the advantages of the two materials, and can also make up for their shortcomings.

### 3.3. Failure Stiffness Modulus

**Figure 9:**
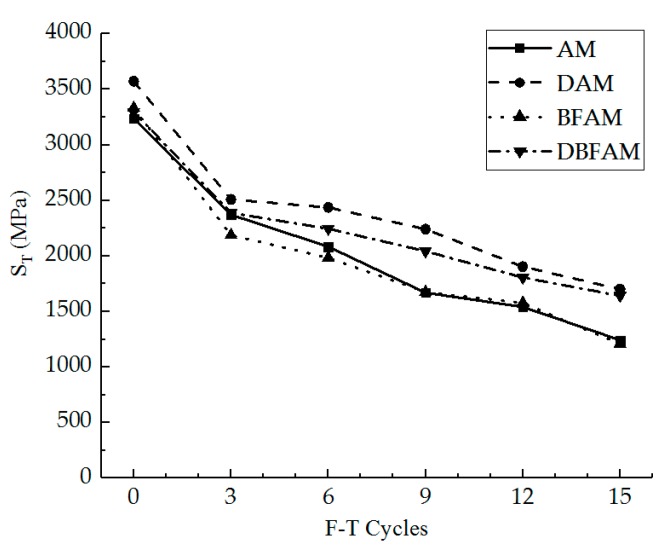
Failure stiffness modulus under F–T cycles.

**Figure 10:**
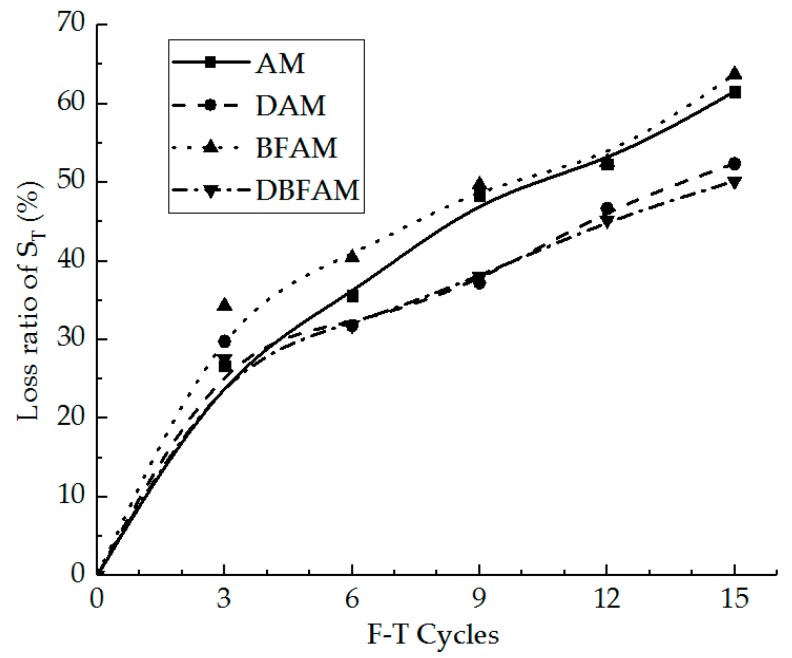
Loss ratio of failure stiffness modulus under F–T cycles.

The failure stiffness modulus and the loss ratio of it were calculated for different specimens under F–T cycles, as shown in Figure 9 and Figure 10.

As can be seen in Figure 9 and Figure 10, the IDT stiffness modulus for each kind of asphalt mixture decreases while the loss ratio increases under F–T cycles. The stiffness modulus of each kind of sample before F–T cycles is similar. The effect of F–T cycles on the stiffness modulus is greater than the modified materials.

### 3.4. Deformation Energy Density

**Figure 11:**
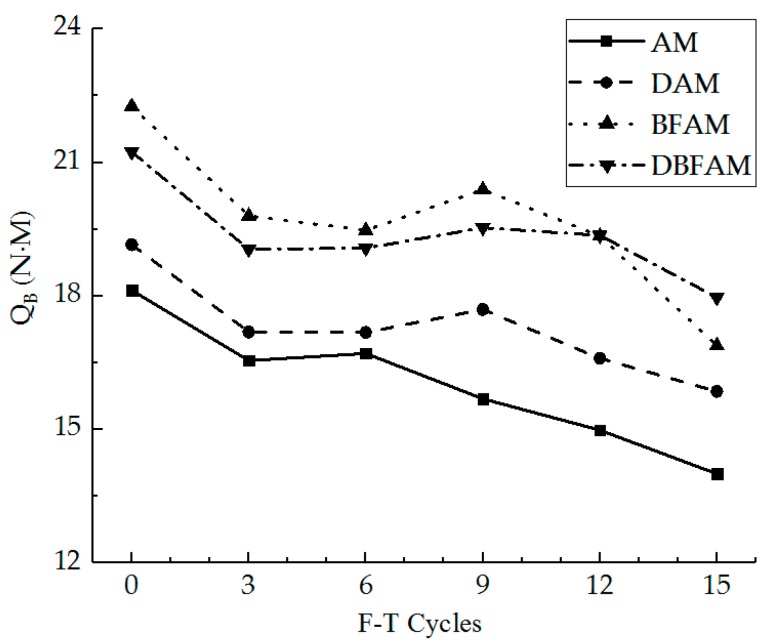
Deformation energy density under F–T cycles.

**Figure 12:**
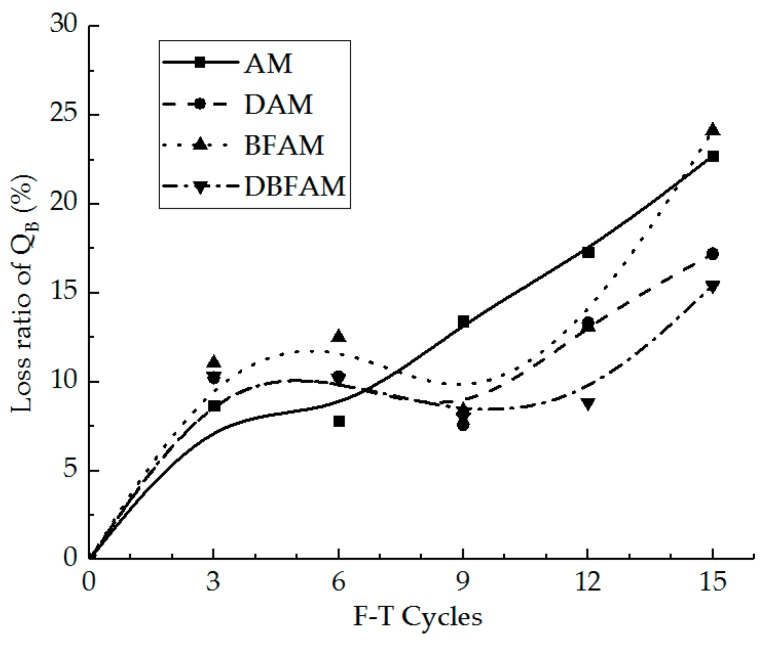
Loss ratio of deformation energy density under F–T cycles.

The deformation energy density and the loss ratio of it were calculated for different specimens under F–T cycles, as shown in Figure 11 and Figure 12.

As can be seen in Figure 11 and Figure 12, the deformation energy density for each kind of asphalt mixture decreases after the F–T cycles. The deformation energy densities of BFAM and DBFAM are higher than that of the AM group. The BFAM has the highest low-temperature crack resistance, while the DBFAM has the second highest resistance. What is more, the loss ratio of the deformation energy density of DAM and DBFAM is smaller than that of the AM group after nine F–T cycles. The DBFAM has the highest F–T cycles resistance while the DAM has the second highest resistance. The deformation energy density result reflects that the compound-modified asphalt mixture can solve the disadvantage of DAM and BFAM, so the DBFAM can be well used in seasonal frozen regions.

Above all, from the indexes changed under F–T cycles, we can see that the low temperature performance after 15 F–T cycles was significant improved by adding both diatomite and basalt fiber. The deformation energy density of DBFAM are 29% higher than AM after 15 F–T cycles, which means the DBFAM pavement can present longer service life in seasonal frozen regions. This is important for reducing the road maintenance and traffic disruption, and the cost expected for AM, BFAM, DAM, DBFAM are 0.330, 0.364, 0.345 and 0.372 CNY/kg, respectively. It is still important in seasonal frozen regions of China although the cost of modified asphalt mixture has increased.

### 3.5. Stress–Strain Curves

**Figure 13:**
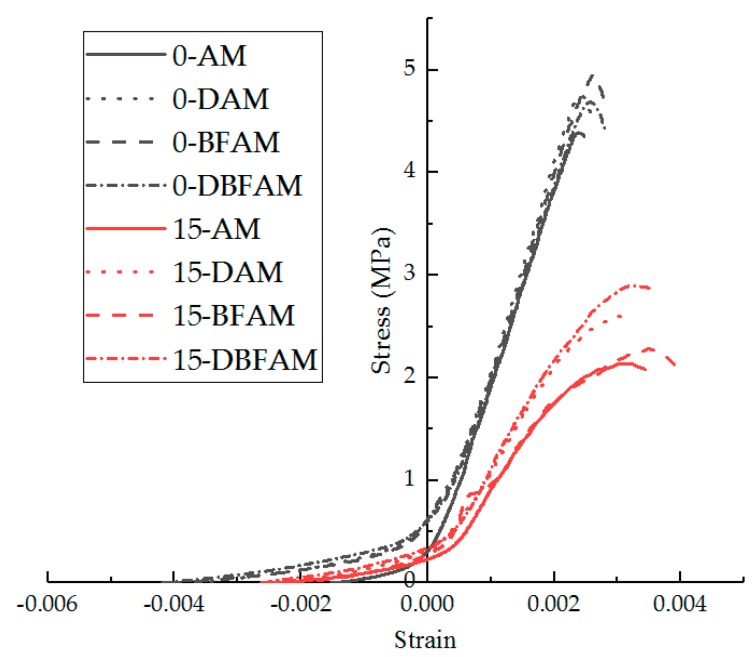
Stress–strain curve of IDT test before and after F–T cycles.

The stress–strain curves were calculated for different specimens under 0 and 15 F–T cycles, as shown in Figure 13.

As can be seen in Figure 13, the stress–strain curve of the asphalt mixture exhibits softening characteristics under the action of F–T cycles. Before the F–T cycles, the IDT test of the asphalt mixture at low temperature is similar to the brittle failure. Once the specimen reaches the failure strain, the bearing capacity is quickly lost. The stress–strain curve is almost linear before the peak point.

After the F–T cycles, the brittleness of the specimens of both groups obviously decreases. The stress–strain curve is nonlinear before the peak point and the nonlinear zone is more significant under F–T cycles. After the peak, it does not completely lose its bearing capacity.

The curve of DAM and DBFAM is above that of AM and BFAM, and their nonlinear zone is less significant than AM and BFAM, which reflects that adding diatomite is effective in enhancing the resistance performance of F–T cycles for asphalt mixtures.

### 3.6. Linear Zone Stress Ratio and Linear Zone Strain Ratio Result

**Figure 14:**
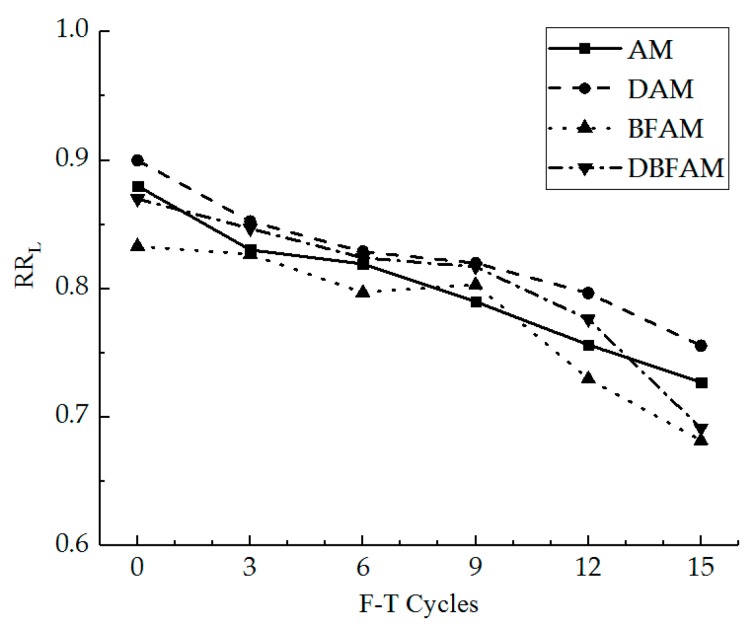
Linear zone stress ratio under F–T cycles.

**Figure 15:**
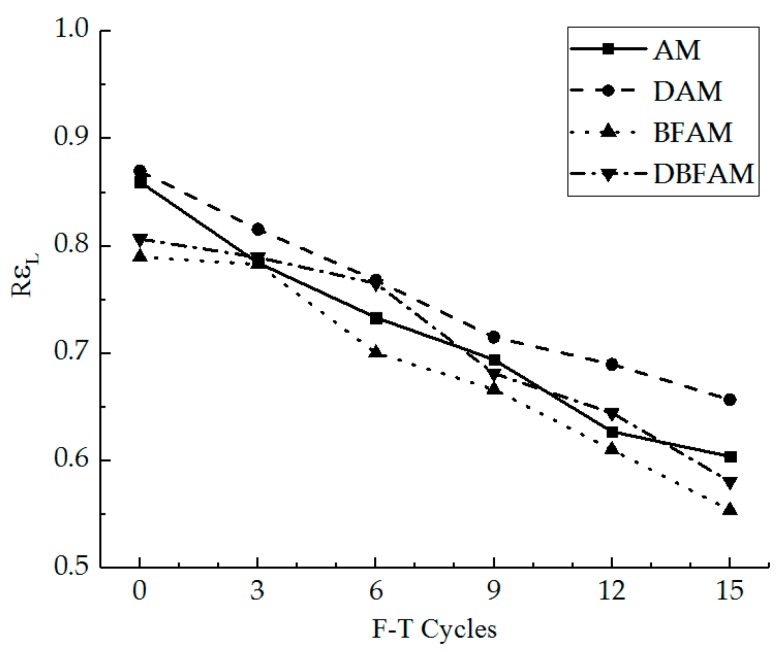
Linear zone strain ratio under F–T cycles.

The peak stress and strain and slope of the stress–strain curve can be described by the strength, strain, and stiffness modulus for the IDT test at low temperature. It seems to treat the samples as elastomers at low temperature. However, it is difficult to reflect the softening characteristics of IDT test at low temperature after F–T cycles. The linear zone stress ratio and linear zone strain ratio are suitable indexes to inflect the nonlinear change of the stress–strain curves. The linear zone stress ratio and linear zone strain ratio were calculated for different specimens under F–T cycles, as shown in Figure 14 and Figure 15.

As can be seen in Figure 14 and Figure 15, in the progress of the F–T cycles, the linear zone stress ratio and linear zone strain ratio decrease for each kind of asphalt mixture. The decreasing trend of the linear zone strain ratio is faster than that of linear zone stress ratio. As the F–T cycle progresses, the range of the nonlinear zone gradually increases in the stress–strain curve, and the nonlinear characteristics of the stress–strain are more significant. The linear zone can reflect the elastic phase of the asphalt mixture at low temperature, while the nonlinear zone reflects the damage stage of crack propagation for the asphalt mixture at low temperature. The initial crack gradually cracks, develops, and penetrates until the specimen reaches the critical cracking point. Under the F–T cycles, the internal crack and air void will increase because of the ice volume expansion [17,33]. The bond force between the asphalt membrane and aggregates declines under the F–T cycles. Thus, it is more difficult to provide a constraint to maintain a linear relationship for the curve in high stress conditions after the F–T cycles, the relative slip happens between the aggregates, and the stress–strain curve changes into the nonlinear zone.

Before the F–T cycles, the linear zone stress ratio and linear zone strain ratio of DAM are the highest, while those of BFAM are the lowest. This indicates that adding basalt fiber can play an anchoring role in the relative slip stage in the failure strain, while adding diatomite is not significant. The IDT failure strain would decrease by adding diatomite and increase by adding basalt fiber. The result for DBFAM reflects that adding basalt fiber can solve the problem of adding diatomite for the asphalt mixture at low temperature by playing the anchoring role in the relative slip stage.

### 3.7. Elastic Stiffness Modulus of Linear Zone Result

**Figure 16:**
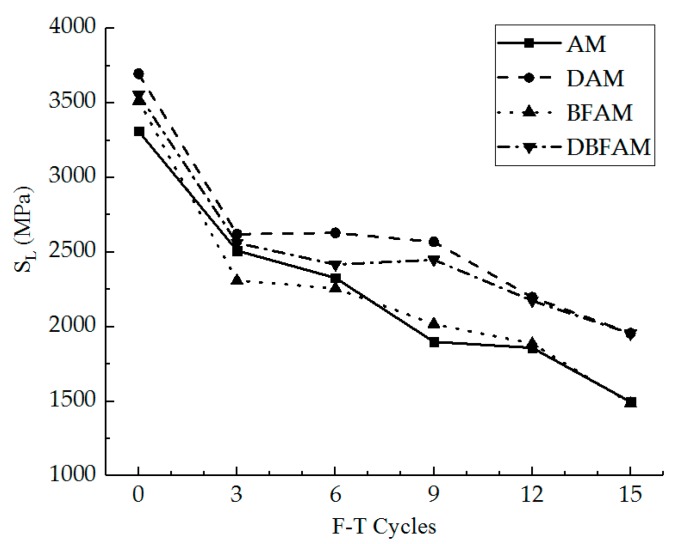
Elastic stiffness modulus of linear zone under F–T cycles.

**Figure 17:**
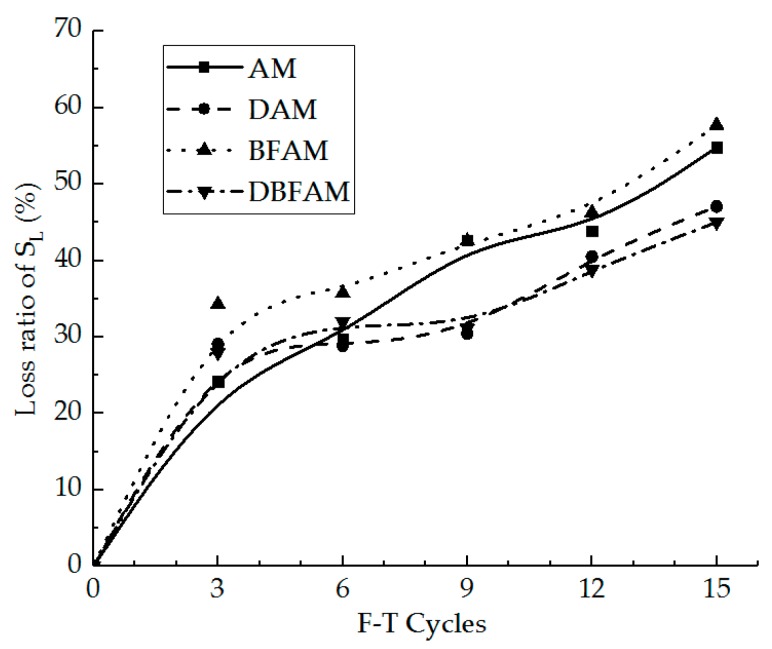
Loss of elastic stiffness modulus of linear zone under F–T cycles.

The elastic stiffness modulus of the linear zone and the loss ratio of it were calculated for different specimens under the F–T cycles, as shown in Figure 16 and Figure 17. The loss ratio of the elastic stiffness modulus is calculated as the damage only caused by the F–T cycles.

As can be seen in Figure 16 and Figure 17, it is similar to the reason for the IDT stiffness modulus in Figure 9 and Figure 10. The elastic stiffness modulus of the linear zone is slightly higher than the IDT stiffness modulus and its loss ratio is also slightly lower than the loss of the IDT stiffness modulus for each kind of asphalt mixture. The loss of the elastic stiffness modulus of the linear zone, which reflects the damage only caused by the F–T cycles, is smaller and smaller than that of the IDT stiffness modulus with the increase of the F–T cycles.

### 3.8. Experiment Verification of Constitutive Model

**Figure 18:**
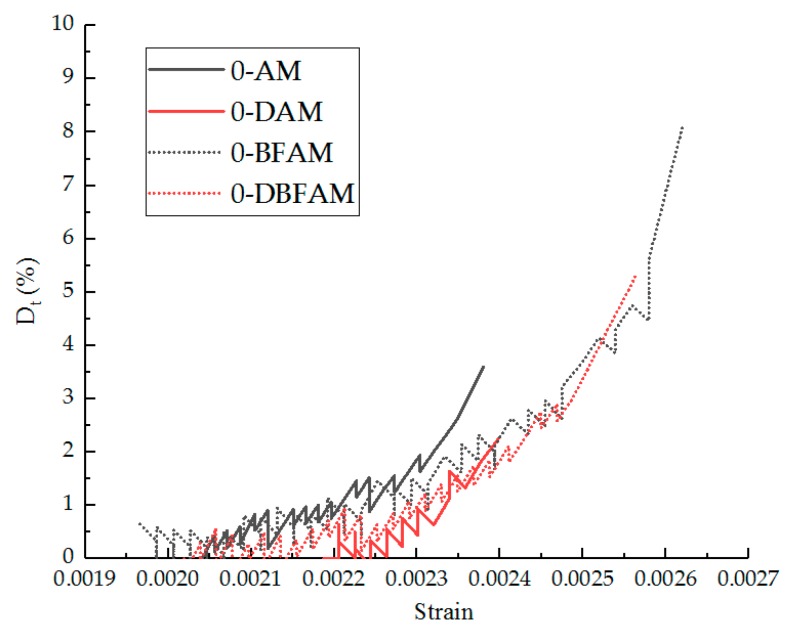
$D_{t}$ values in the nonlinear zone before F–T cycles.

**Figure 19:**
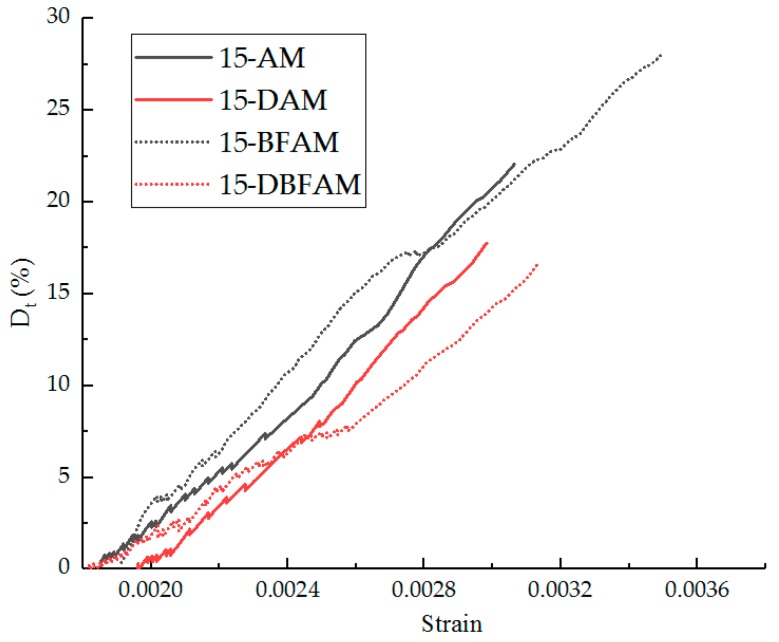
$D_{t}$ values in the nonlinear zone after 15 F–T cycles.

The $D_{t}$ values in the nonlinear zone can be calculated according to Equation (12). The $D_{t}$ values curves of the nonlinear zone for each group of asphalt mixture before and after 15 F–T cycles are shown in Figure 18 and Figure 19, respectively.

According to the analysis of the damage curve, the difference of the damage degree curve is obvious before and after the 15 F–T cycles, the damage initiation stage is moved forward, and the damage degree is also obviously increased. The nonlinear zone before the peak increases significantly, which reflects the softening effect of the F–T cycle, and the nonlinearity after the F–T cycles is more significant. After the basalt fiber is added, the nonlinear zone also has obvious growth, which reflects the toughening and crack-preventing effect of the fiber. This is consistent with the effect of the two nonlinear indicators, the linear zone stress ratio and the strain ratio.

**Figure 20:**
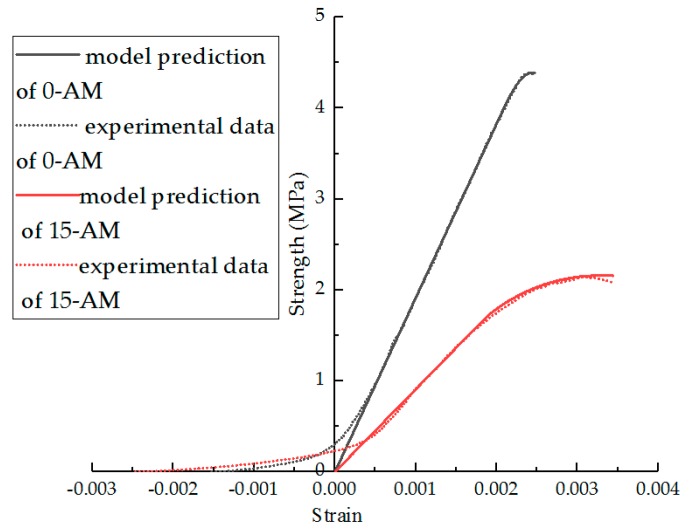
Comparison of experimental data and model predictions for AM under F–T cycles.

**Figure 21:**
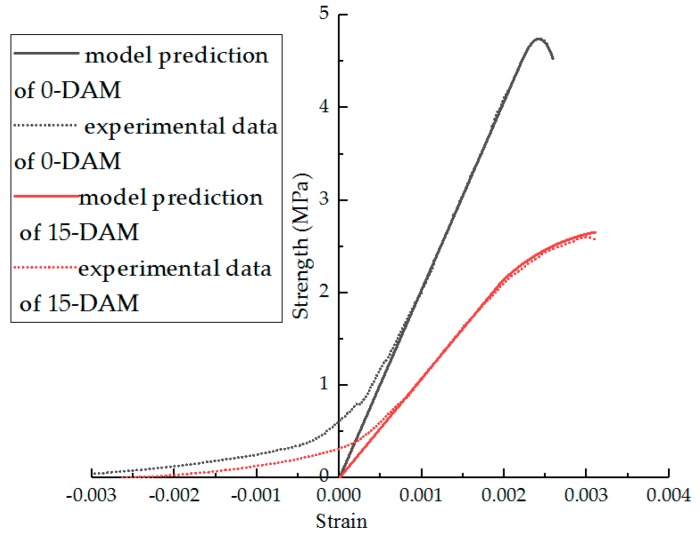
Comparison of experimental data and model predictions for DAM under F–T cycles.

**Figure 22:**
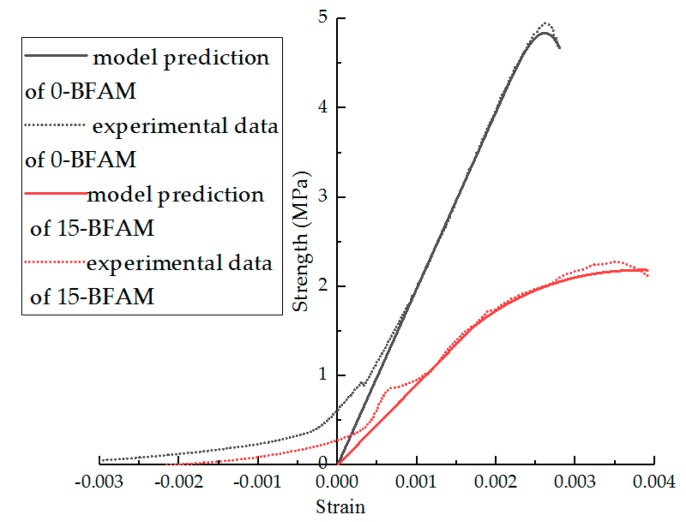
Comparison of experimental data and model predictions for BFAM under F–T cycles.

**Figure 23:**
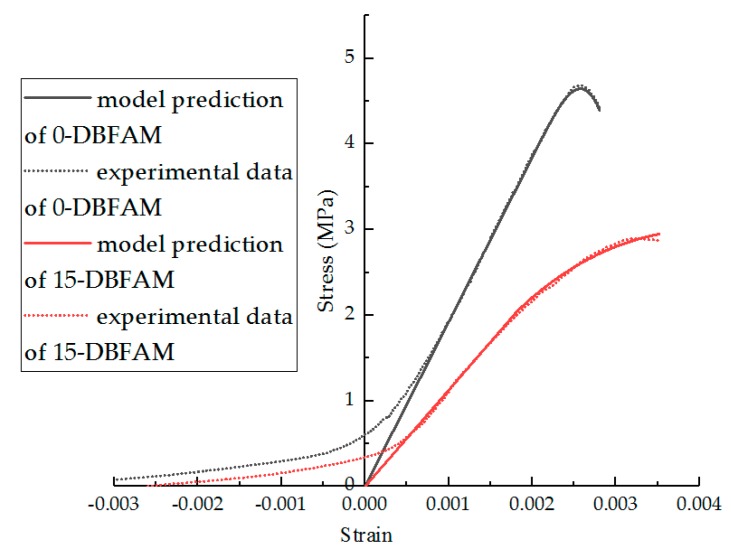
Comparison of experimental data and model predictions for DBFAM under F–T cycles.

From Equation (14), the parameters of the model can be fitted, and the results are shown in Table 6. In Table 6, the effect of the F–T cycles on parameters m and n is obviously higher than that of modification. The F–T cycle makes the parameter m decrease and n increase. The R^2^ indicates that the statistical damage constitutive model established by Equation (15) can better describe the stress–strain relationship after F–T damage. Comparisons of the experimental results and statistical damage constitutive model predictions of different asphalt mixture are shown in Figure 20, Figure 21, Figure 22 and Figure 23. The results show that the statistical damage constitutive model is suitable for different asphalt mixtures at low temperature.

## 4. Conclusions

In this research, the low-temperature indirect tensile test was used to investigate the variation of low-temperature performance of the asphalt mixture under F–T cycles. The influence of adding basalt fiber and diatomite on the asphalt mixture for the low-temperature properties and resistance of F–T cycles was analyzed by many indexes. We described the variation of the stress–strain curve under F–T cycles and proposed a statistical damage constitutive model under F–T cycles and loading for asphalt mixtures. From this research, the following conclusions can be drawn:

- F–T cycles will reduce the IDT strength, stiffness modulus and strain energy density of asphalt mixture, and increase the IDT failure strain. As the number of F–T cycles increases, the variation of each index decreases.
- After adding basalt fiber, the IDT strength, IDT failure strain, and strain energy density of the mixture are improved, and the low-temperature performance is improved. After adding diatomite, the loss ratio of each index decreases under F–T cycles, and the resistance of F–T cycles is improved. The low-temperature performance and resistance of F–T cycles for asphalt mixture are improved after compound modified by basalt fiber and diatomite. The eco–friendly basalt fiber–diatomite-modified asphalt mixture is suitable in seasonal frozen regions.
- The variation law and form of the stress–strain curve before and after the F–T cycle are proposed. The stress–strain curve is divided into linear zone and nonlinear zone. Under the action of the F–T cycles, the stress ratio in the linear zone is gradually reduced, the strain ratio in the nonlinear zone is gradually increased, and the nonlinear characteristics of the stress–strain are more significant. After the addition of basalt fiber, the nonlinear zone increases significantly, which reflects the reinforcement of the fiber during the cracking stage. The nonlinear point of the stress–strain curve can be described by the nonlinear index, and the mechanism of freezing and thawing and the mechanism of material modification are better reflected.
- The statistical damage constitutive model established in this paper can describe the stress–strain relationship of the asphalt mixture at low temperature after F–T cycles well.

## Figures and Tables

**Table 1:** Technical parameters of tested asphalt.

Property	Value	Standard
Density (15 °C, g/cm^3^)	1.018	-
Penetration (25 °C, 0.1 mm)	92.3	80~100
Softening point *T*_R&B_ (°C)	46.9	≥42
Ductility (25 °C, cm)	>150	≥100
Viscosity (135 °C, mPa·s)	306.9	-

**Table 2:** Properties of aggregate.

Sieve size (mm)	13.2	9.5	4.75	2.36	1.18	0.6	0.3	0.15	0.075
Apparent density (g/cm^3^)	2.811	2.805	2.815	2.817	2.808	2.805	2.778	2.777	2.768
Absorption coefficient of water (%)	0.33	0.44	0.54	0.75	-	-	-	-	-

**Table 3:** Physical properties of mineral powder.

Property	Hydrophilic Coefficient	Apparent Density (g/cm^3^)	Gradation	
			Sieve Size (mm)	Passing (%)
Value	0.778	2.722	0.6	100
			0.15	95
			0.075	80

**Table 4:** Properties of diatomite.

Property	Color	Bulk Density	Specific Gravity	pH
Value	White	0.38 g/cm^3^	2.1 g/cm^3^	7

**Table 5:** Properties of basalt fiber (provided by manufacturer).

Items	Value	Standard Value
Diameter (µm)	10–13	-
Length (mm)	6	-
Water content (%)	0.030	≤0.2

**Table 6:** The parameters of the model.

F–T	Parameters	AM	BFAM	DAM	BFDAM
0	m	2.47	3.54	2.48	3.36
	n	8.13 × 10^−4^	9.33 × 10^−4^	5.93 × 10^−4^	9.09 × 10^−4^
	R^2^	0.935	0.983	0.997	0.999
15	m	1.33	1.2	1.27	1.28
	n	2.73 × 10^−3^	3.50 × 10^−3^	3.00 × 10^−3^	3.69 × 10^−3^
	R^2^	0.995	0.992	0.997	0.982
